# The swiss neonatal quality cycle, a monitor for clinical performance and tool for quality improvement

**DOI:** 10.1186/1471-2431-13-152

**Published:** 2013-09-28

**Authors:** Mark Adams, Tjade Claus Hoehre, Hans Ulrich Bucher

**Affiliations:** 1 Division of Neonatology, University Hospital Zurich, Zurich, Switzerland

**Keywords:** Very preterm infants, Very low birth weight infants, Quality assessment, Quality indicators, Benchmarking, Falsification, Mortality, Morbidity, Evidence based medicine

## Abstract

**Background:**

We describe the setup of a neonatal quality improvement tool and list which peer-reviewed requirements it fulfils and which it does not. We report on the so-far observed effects, how the units can identify quality improvement potential, and how they can measure the effect of changes made to improve quality.

**Methods:**

Application of a prospective longitudinal national cohort data collection that uses algorithms to ensure high data quality (i.e. checks for completeness, plausibility and reliability), and to perform data imaging (Plsek’s p-charts and standardized mortality or morbidity ratio SMR charts). The collected data allows monitoring a study collective of very low birth-weight infants born from 2009 to 2011 by applying a quality cycle following the steps ′guideline – perform - falsify – reform′.

**Results:**

2025 VLBW live-births from 2009 to 2011 representing 96.1% of all VLBW live-births in Switzerland display a similar mortality rate but better morbidity rates when compared to other networks. Data quality in general is high but subject to improvement in some units. Seven measurements display quality improvement potential in individual units. The methods used fulfil several international recommendations.

**Conclusions:**

The Quality Cycle of the Swiss Neonatal Network is a helpful instrument to monitor and gradually help improve the quality of care in a region with high quality standards and low statistical discrimination capacity.

## Background

In Switzerland, as in many other countries, participating in a quality assessment collaborative has recently become mandatory for all intensive care units. As a neonatology unit’s patients cannot be compared with the average intensive care patient, the Swiss Society of Neonatology decided to design its own approach to quality assessment. In 2006 it started with developing standards for the quality of care of new-borns. The meanwhile implemented standards oblige the Swiss neonatology units to fulfil requirements regarding staffing, equipment and to apply evidence based protocols in order to be classified into the internationally recognized levels of neonatal care I – III [1]. At the third and top level, units are required to participate in the Swiss Neonatal Network. The Swiss Neonatal Network prospectively records standardized data for all children born alive between a gestational age of 23 0/7 to 31 6/7 weeks or a birth weight below 1501 g, all children as of 32 weeks gestational age requiring continuous positive airway pressure (CPAP), all children with perinatal encephalopathy requiring therapeutic hypothermia, and follow-up data of selected high-risk collectives at two and five years corrected age. The collected data is used for research on the one hand (see for example [2,3]) and for quality assessment on the other. For the latter, the network has devised a quality assessment tool based on recent peer-reviewed findings and reviews that comment on the proper use and efficacy of quality improvement initiatives in medicine.

In this publication we describe the setup of this tool and list which requirements it fulfils and which it does not. We report on the so-far observed effects and how the units can monitor the effect of changes made in the clinic to improve quality where appropriate. We describe how the tool functions like a bed-side monitor where the clinic is the patient under observation and the network’s tool is the monitor that provides constant feed-back to the clinicians and alerts them if and when their clinic’s data moves out of range. Finally, we propose that this setup approaches the yet to be established requirement for evidence based medicine to continuously test its own hypothesis.

## Methods

### Study collective

For the purpose of this study we limit our collective to all live-born infants (including patients that died in the delivery room) born between 501 to 1500 g birth-weight as this is the best described collective of preterm children and provides the most data for benchmarking comparisons. Data was collected from 2006 to 2011 by all nine level III neonatal intensive care units either via exporting data from their clinical information system (4 NICUs) and subsequent import into the national database or via direct data entry into the national database (5 NICUs). 98 items were collected for all live-born children from birth until death or first discharge home. 30 items were collected for all children that died in the delivery room. All items are defined in a manual [4]. They cover typical aspects of perinatal care, demographics, common diagnoses and treatments, growth and hospitalization duration.

Data collection and evaluation for this study were approved by the Swiss Federal Commission for Privacy Protection in Medical Research. Participating units were obliged to inform parents about the scientific use of the anonymized data.

### Data item selection

Out of the 98 items collected, a group of experts selected those items that reflect the performance of the individual units as opposed to items that cannot be modulated (such as gender, birth defects, socio-economic status, etc.). The selected items fulfil international standards for the description of mortality and common morbidities in very low birth-weight children [5,6].

The selected items were then tested for their suitability as quality indicators (QI’s) using the strict criteria of QUALIFY [7]. QUALIFY was developed by the German National Institute for Quality Measurement in Health Care (BQS) as an instrument for the structural appraisal of quality indicators in health care. It offers 3 criteria for the proposed quality indicator’s relevance, 8 for its scientific soundness, and 9 for its feasibility.

### Data processing and imaging

**Figure 1:**
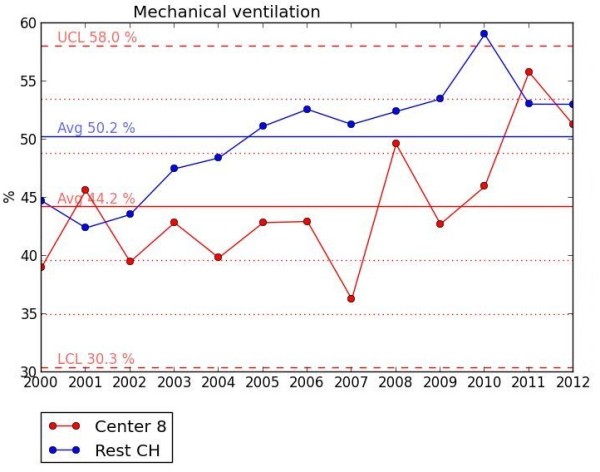
**Benchmarking diagram.** Plsek’s p-chart for mechanical ventilation for unit 8 versus the other level III NICUs in Switzerland (CH) displaying historical annual percentages for 2000–2012. The mean (Avg) percentage over the entire period is 44.2% for the unit and 50.2% for CH. The 1st and 2nd standard deviation (SD) of the unit are dotted lines (SD were calculated using the formula SD = SQRT {[mean percentage x (1 - mean percentage)] / [sample size]}) whereas the 3rd SD are dashed lines. The unit’s upper and lower control limits (UCL = 58% and LCL = 30.3%, respectively) are set by convention at ± 3 SD beyond the mean.

Benchmarking diagrams (Figure 1): For the identification of problematical areas, python-scripts (using matplotlib [8]) extract and evaluate the network data over night and display the results in one Plsek’s p-chart per item per unit accompanied by a table with information on collective size, effect size and number of missing entries [9,10]. In our setting, Plsek’s p-chart displays the effect size of an item over time with one dot per year for the given unit versus the rest-collective. Horizontal lines reflect the mean rate over time, one for the unit and one for the rest-collective, respectively, as well as one each for the unit’s first, second and third standard deviation of the mean value. Crossing the third standard deviation of the mean in any given year is considered a significant change.

**Figure 2:**
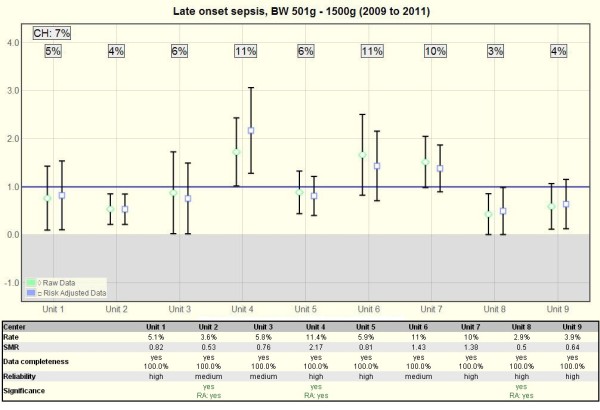
**Quality indicator chart.** Example QI-chart (Late onset sepsis) with a diagram above and a table below. The diagram is based on the standardized mortality / morbidity ratio model and compares each unit (1–9) with the combination of all level III NICUs in Switzerland (CH). The rate of the entire collective (CH) is set as 1 and is compared with the unit’s observed relative raw rate (diamond) or its risk-adjusted (currently only gestational-age adjusted) observed vs. expected rate (square). A missing overlap of a 95% confidence interval marks a significant difference between a unit and the entire community. The table below lists the detailed rate, SMR, data completeness, reliability and whether the difference is significant (as this is not always clearly visible in the diagram). The rate of the entire collective (CH) is in the top left corner of the diagram.

Quality indicator diagrams (Figure 2) are generated after the finalization of a year’s data collection using python-scripts for the calculation and javascript/jquery for the presentation of the data. The diagrams are based on the standardized mortality or morbidity ratio (SMR) model [11] in which the entire collective is set as 1 and the unit’s value per item is displayed in relation to the collective value with a 95% confidence interval. Below the diagram, each value is commented upon in a table listing information on unit rate, SMR value, data completeness and reliability. There are two sets of diagrams, one (Figure 2) displaying one item per diagram with the nine units de-identified side by side in a row, and one (not shown) displaying a selection of items per unit so that the possible effect of one item upon another can be observed. Outcome quality indicators (as opposed to process quality indicators) display both the unadjusted and the risk-adjusted values next to each other. Risk-adjustment is based on the units’ individual distribution of children into the gestational age groups below 24, 24–25, 26–27, 28–29, 30–31, and above 31 weeks.

### Data quality

Upon entry into the national database, every record is checked for data completeness and plausibility. Data deemed as erroneous by the system are subject to be corrected by the participating units.

The data collection is compared annually to the birth registry of the Swiss Federal Statistical Office to ensure record completeness.

Those items subject to the QUALIFY quality indicator requirements are additionally checked for measurement completeness, reliability and discrimination capacity:

Measurement completeness: Items to which the network receives less than 90% answers from any given unit are excluded from evaluation for the respective unit. The degree of completeness is displayed in percentage per unit below each diagram.

Reliability: Assuming that health care changes are gradual as opposed to erratic, the quality indicator is analysed for change over time. For this analysis, the QI in question is scrutinized for the period of interest (2009–2011) and the same time period in advance (2006–2008) for each unit separately: The combined time period (2006–2011) is split into eight sections and the development of the QI is monitored over time by plotting the QI’s rate and 95% confidence interval side by side for each of the 8 sections. If the confidence intervals of two neighbouring sections do not overlap, an erratic change is assumed and the data of this unit for this QI is deemed only partially reliable. If the intervals do not overlap twice or more, the data from this unit is deemed unreliable and is excluded from further evaluation. If a section appears at a rate of 0% or 100% and the confidence intervals therefore equal 0, no erratic change is assumed and the next section is compared with the rate of the previous section that was different from 0% or 100%. The exact degree of reliability is displayed below each diagram.

Discrimination capacity: statistical discrimination capacity is optimized by the pooling of years and by monitoring data completeness. A difference between a participating unit and the entire collective is considered significant when the 95% confidence interval of the unit does not overlap 1.

### Quality cycle

Upon password protected login, the unit’s representative can browse his/her unit’s data, error and missing lists and evaluations. Twice per year, the representatives meet to discuss results.

**Figure 3:**
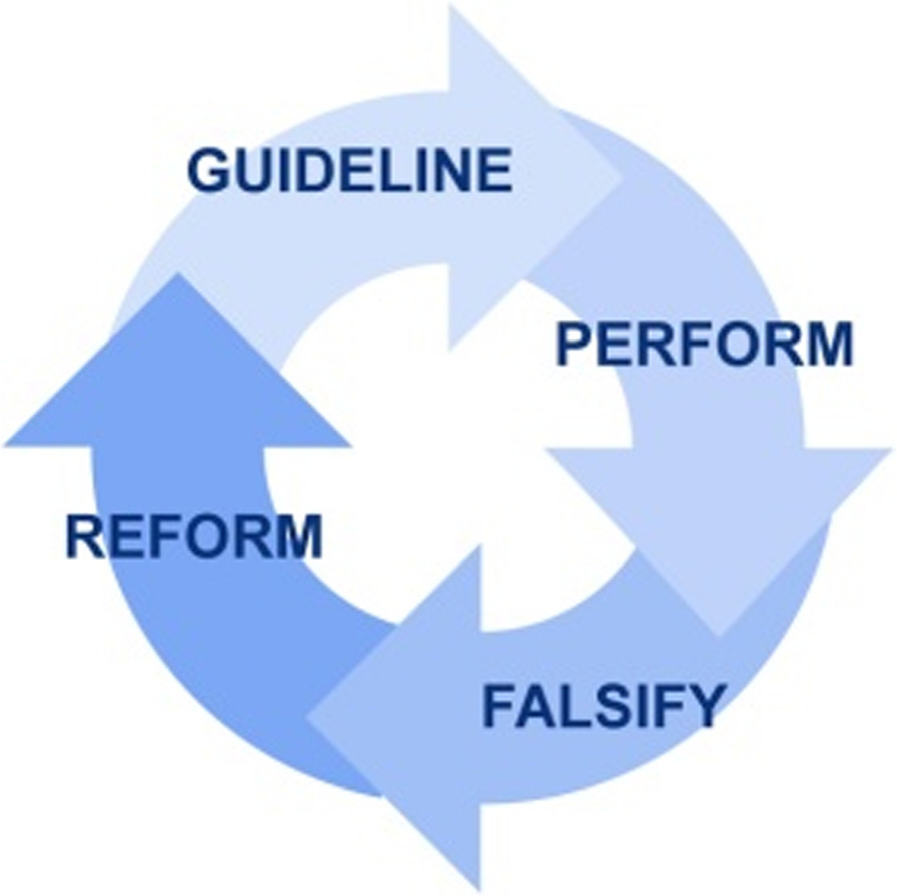
**Quality cycle.** Quality cycle of the Swiss Neonatal Network.

This final step completes the quality cycle (Figure 3): Swiss level III neonatology units apply evidence based written protocols for medical and nursing staff and standard operating procedures for the collaboration with obstetricians and other paediatric subspecialties (*Guideline*) [1]. The guidelines are used in every day clinic (*Perform*) while maintaining a Critical Incident Reporting System (CIRS). Process and outcome are constantly monitored using the above described data processing tools in order to locate possible progress and setbacks (*Falsify*). At the biannual meetings the results are discussed and change in individual units or at the level of the network are initiated (*Reform*).

The meetings are setup such that two to three quality indicators with noteworthy values (i.e., large differences between units, large difference between Swiss data and published international data or large difference over time) are chosen for the subsequent meeting and given to individual unit directors for analysis. At the subsequent meeting, the values for these quality indicators and their most likely causes for difference according to Pareto [10] are presented. The plenum then discusses changes that are expected to lead to improvement. If a conclusion cannot be reached due to lack of time, missing extra analysis or references, the discussion can be continued in an online forum. If a change is made, the effect of the change will be measured and scheduled for discussion at a subsequent meeting. On-going data collection is planned in order to secure long-term improvement.

Falsification: The concept of Falsification was developed by Sir Karl Popper, an important philosopher of science of the 20th century. Popper is known for his attempt to repudiate the classical observationalist/inductivist form of scientific method in favour of empirical falsification. According to Popper, a theory should be considered scientific if, and only if, it is falsifiable. He considers science to be “a critical activity. We test our hypotheses critically. We criticize them to find mistakes; and in hope to eliminate the mistakes and so come closer to the truth” [12].

### Statistical analysis

For this publication, two-sided Mann–Whitney U-tests were performed to compare mean values of two independent variables. To determine differences in the distribution of a variable, the Pearson’s Chi-square test was used. Probability levels below 0.05 were considered significant. Statistical analyses were carried out with Python release 2.7 using matplotlib and Microsoft Excel 2011.

## Results

The 9 Level III neonatology units of the Swiss Neonatal Network registered 2025 live-births with a birth weight between 501 to 1500 g from 2009 to 2011 (Table 1). They represent 96.1% of all very low birth-weight live-births in Switzerland according to the birth registry of the Swiss Federal Statistical Office [13] (96.2% for 2009, 96.3% for 2010 and 96.0% for 2011). The number of children per unit range from 95 to 388 for the pooled 3 year period. A comparison to the rates of the Vermont Oxford Network [14] shows that the population is not significantly different as far as gender distribution and rate of children ′small for gestational age′ is concerned. The rate of multiple births however is significantly higher in Switzerland. Concerning the outcome, the mortality is not significantly different, whereas several important morbidities (PDA, NEC, late onset sepsis, oxygen at 36 weeks gestational age, ROP stage 3–4, and PIH stage 3–4) are lower in Switzerland.

**Table 1:** Data analysis

	**Swiss neonatal network**			**Vermont-oxford-network**	**EuroNeoNet**	**Difference VON-SNN**
	All units	min.	max.			
Years	2009-2011	2009	2011	2010	2010	-
Units	9	-	-	ca. 850	96	-
N	2025	95	388	53862	6389	-
Sex male	52.2%	42.3%	59.8%	51.0%	50.4%	0.29
Multiples	36.0%	24.9%	44.2%	28.0%	33.1%	<0.001
Small for Gestation	19.2%	15.4%	21.4%	21.0%		0.05
Inborn	95.0%	91.5%	97.8%	86.0%		<0.001
UapH measured	82.7%	*64.4%	*94.7%			
Caesarean Section	77.2%	*63.4%	*88.5%	73.0%	72.3%	<0.001
Full prenatal steroids	71.1%	*59.8%	*79.8%			
Any prenatal steroids	87.9%	79.7%	*98.6%	78.0%	83.5%	<0.001
Major birth defects	5.0%	3.4%	7.0%	5.0%		1
Mortality	13.6%	5.8%	*19.4%	12.6%	10.8%	0.185
PDA†	28.3%	*11.2%	*41.8%	37.0%		<0.001
PPH†	5.4%	*0.9%	*12.4%			
NEC†	2.0%	0.6%	3.6%	6.0%	5.9%	<0.001
Early Onset Sepsis†	2.0%	0.6%	*7.4%	2.0%	3.5%	1
Late Onset Sepsis†	7.4%	2.5%	*11.7%	15.0%	25.5%	<0.001
O2 at 36w GA‡	9.5%	3.0%	19.8%	30.0%	10.7%	<0.001
Mechanical ventilation†	51.8%	*40.6%	*71.5%	64.0%		<0.001
CPAP†	78.4%	*67.3%	*86.3%	69.0%		<0.001
CPAP w/o mech. vent.†	31.3%	*12.6%	*47.6%			
Surfactant†	45.9%	*25.1%	*65.2%	64.0%	51.8%	<0.001
ROP stage 3-4†	2.3%	0.0%	5.3%	6.0%	2.7%	<0.001
PIH stage 3-4†	6.4%	3.8%	9.8%	9.0%	7.7%	<0.001
cPVL†	2.3%	0.0%	4.7%	3.2%	3.9%	0.027
Growth†	9.4%	*7.7%	*14.5%			
Surgery†	10.3%	5.0%	15.2%	16.0%		<0.001
Length of stay†	56.9	46.9	65.2			
Length of stay‡	55.0	44.2	64.4	64.8		n/a

Data analysis and comparison to Vermont-Oxford-Network [14] and EuroNeoNet [15] for all live-births between 501-1500 g birth-weight (without delivery room deaths (†), or that have survived until discharge home (‡)). For a definition of the listed items see [2,4]. The first column shows the mean Swiss value. The second and third columns render the lowest (min.) and highest (max.) value achieved by one of the network units. An asterisk (*) marks where each value significantly differs from the value of the entire Swiss collective. UapH: umbilical artery pH, PDA: patent ductus arteriosus, PPH: positive pulmonary hypertension, NEC: necrotizing enterocolitis, CPAP: continuous positive airway pressure, ROP: retinopathy of prematurity, PIH: periventricular- intraventricular haemorrhage, cPVL: cystic periventricular leucomalacia, growth: rate of children born small for gestational age that have surpassed the 10th weight percentile by discharge.

Differences between the individual Swiss units, of which the lowest (min.) and highest (max.) value are shown in Table 1, are surprisingly large and in many cases result in a confirmed significance (* in Table 1).

From the 24 variables used for the evaluation of unit to unit differences (Table 2), 23 are available as benchmarking diagrams (Figure 1) and 20 as quality indicator diagrams (Figure 2), thereof 5 for process and 15 for outcome indicators.

**Table 2:** Data quality

	**Bench-marking**	**Quality indicators**	**Data completeness**			**Reliability**
			All units	min.	max.	
Inborn	yes	yes (P)	99.6%	96.3%	100.0%	8 / 1 / 0
UapH measured	no	yes (P)	100.0%	100.0%	100.0%	6 / 3 / 0
Caesarean Section	yes	yes (P)	100.0%	100.0%	100.0%	4 / 4 / 1
Full prenatal steroids	yes	yes (P)	96.3%	90.8%	99.7%	5 / 3 / 1
Any prenatal steroids	yes	yes (P)	96.3%	90.8%	99.7%	6 / 3 / 0
Major birth defects	yes	no	100.0%	100.0%	100.0%	-
Mortality	yes	yes (O)	100.0%	100.0%	100.0%	6 / 3 / 0
PDA	yes	yes (O)	100.0%	100.0%	100.0%	6 / 3 / 0
PPH	yes	yes (O)	100.0%	100.0%	100.0%	7 / 2 / 0
NEC	yes	yes (O)	100.0%	100.0%	100.0%	8 / 1 / 0
Early Onset Sepsis	yes	yes (O)	100.0%	100.0%	100.0%	8 / 1 / 0
Late Onset Sepsis	yes	yes (O)	100.0%	100.0%	100.0%	6 / 3/ 0
O2 at 36w GA	yes	yes (O)	97.8%	91.6%	100.0%	6 / 3 / 0
Mechanical ventilation	yes	yes (O)	100.0%	100.0%	100.0%	6 / 3 / 0
CPAP	yes	yes (O)	100.0%	100.0%	100.0%	4 / 4 / 0
CPAP w/o mech. vent.	yes	yes (O)	100.0%	100.0%	100.0%	6 / 2 / 1
Surfactant	yes	yes (O)	100.0%	100.0%	100.0%	7 / 1 / 1
ROP stage 3-4	yes	yes (O)	83.2%	72.4%	95.8%	9 / 0 / 0
PIH stage 3-4	yes	yes (O)	98.9%	97.7%	100.0%	8 / 1 / 0
cPVL	yes	yes (O)	98.9%	97.7%	100.0%	9 / 0 / 0
Growth	yes	yes (O)	97.9%	92.7%	100.0%	6 / 3 / 0
Surgery	yes	no	100.0%	100.0%	100.0%	-
Length of stay	yes	no	98.7%	90.5%	100.0%	-
Length of stay	yes	no	98.6%	90.0%	100.0%	-

Variables chosen for benchmarking (yes/no) and quality indicator evaluation (yes/no) with their completeness and reliability. (P): process quality indicator (O): outcome quality indicator. Data completeness is given as mean value for all units and with the value of the unit representing the minimum and the maximum completeness. Data is categorized according to the number of units with reliable / partially reliable / unreliable data.

Data completeness in general is high. In some areas there is an improvement potential, for instance in the variables of prenatal steroids, oxygen at 36 weeks gestational age, growth, and length of stay. The low data completeness for ROP 3–4 reflects the fact that many units in Switzerland have ceased to screen children for ROP above 31 weeks gestational age.

Reliability should be tested for those units whose data was calculated as being unreliable: 1 unit for caesarean section, 1 for full prenatal steroids, 1 for CPAP w/o mech. vent., and 1 for surfactant. The reliability testing system of the network is somewhat prone to produce false negative results because of the small size of some of the participating units. If high data reliability can be verified by review of the original case documentation, the testing system can be manually overridden.

Of the twenty criteria required for quality indicators according to QUALIFY [7], the network applies fourteen as instructed and three in a modified version (Table 3). The remaining three criteria are omitted as incompatible.

**Table 3:** List of QUALIFY criteria

**QUALIFY criteria**		**SNN**
**Relevance**	Importance of the quality characteristic captured with the quality indicator for patients and the health care system	applied
	Benefit	applied
	Consideration of potential risks / side effects	applied
**Scientific soundness**	Indicator evidence	applied
	Clarity of the definitions (of the indicator and its application)	applied
	Reliability	modified
	Ability of statistical differentiation	modified
	Risk adjustment	modified
	Sensitivity	-
	Specificity	-
	Validity	applied
**Feasibility**	Understandability and interpretability for patients and the interested public	-
	Understandability for physicians and nurses	applied
	Indicator expression can be influenced by providers	applied
	Data availability	applied
	Data collection effort	applied
	Barriers for implementation considered	applied
	Correctness of data can be verified	applied
	Completeness of data can be verified	applied
	Complete count of data sets can be verified	applied

List of the QUALIFY criteria developed by the German National Institute for Quality Measurement in Health Care (BQS). SNN: Swiss Neonatal Network.

The Quality cycle of the network also fulfils all requirements made by the Swiss Academy of Medical Sciences [16] with the exception that it does not meet the standard of having the data independently externally audited.

In order to identify possible areas of quality improvement, the network members apply a pre-defined procedure: Using benchmarking diagrams, units can identify problematical areas by observing the development of their raw data over time. Using quality indicator diagrams, a suspected problem can be verified under more controlled conditions for a given time period. The thus identified problem is presented and discussed at the bi-annual meeting of the units’ directors and strategies for improvement are sought. After implementation at the clinic, the Plsek’s p-charts finally allow the unit to observe the effect of a change made in the clinic with up to date values of the unit.

So far, the network’s data processing and quality cycle has allowed the revision of the Swiss Neonatal Society‘s guidelines for perinatal care at the limit of viability in 2011 [17] where the recommended gestational age for engaging into intensive care was lowered from 25 to 24 weeks [18]. It has also lead to the replacement of hand disinfectant in one of the participating units and to the revision of oxygen saturation levels in all Swiss Level III units.

## Discussion

### Identification of improvement areas

In the field of neonatology there are no available gold standards in the sense of “best available test or benchmark under reasonable conditions”. It is therefore difficult to define good quality. Instead, one has to rely on the comparison between units which is prone to bias because not all units work under the same conditions. Some have a higher risk for mortality or morbidities than others because of the nature of the collective they treat. We therefore believe that a comparison should not classify a unit with such crude a label as performing with good or bad quality. Instead, we propose a concept where units performing worse in areas where others excel can profit from the latter and improve their quality without losing face. It helps that the detection tool is sensitive enough to show that every unit has areas to improve and that Switzerland is small enough for all participants to know each other well. We have thus adopted two important aspects of the Vermont Oxford Network’s innovative NICQ system where a small number of units respectfully help each other by objectively communicating their results and holding themselves accountable [11].

Areas where at least one Swiss unit differs significantly from the combined Swiss total and which thus display improvement potential lay in the rates of caesarean section, prenatal steroids, mortality, early onset sepsis, late onset sepsis, growth and measured UapH. Berger et al. (2012) previously reported that factors other than baseline population demographics or differences in the interpretation of national recommendations (for children born at the limit of viability) influence survival rates of extremely preterm infants in the individual units which also suggests the presence of areas for improvement [18].

Differences between PDA, PPHN, mechanical ventilation, CPAP, CPAP without mechanical ventilation, and surfactant usage on the other hand are suspected to be reflections of clinical treatment strategy, different diagnostics or geographical location and are therefore of limited use for quality assessment. Yet they can be important when investigating the most likely cause of a quality problem in another measurement.

### Quality of data set

The variables used for the benchmarking and quality indicator calculations were chosen because of their capacity to describe clinically important and/or modifiable processes and outcome [11]. Many of them also appear in the Baby-MONITOR, a composite indicator for quality recently published by Profit et al. (2011) that both an expert panel as well as practicing clinicians agreed upon as having high face validity [19,20]: prenatal steroids, late onset sepsis, oxygen at 36 weeks postmenstrual age, growth velocity and in-hospital mortality. However, in order to complete Profit et al.’s choice of measures included into the Baby-MONITOR, the network would need to add timely ROP exam, pneumothorax, human milk feeding at discharge and hypothermia on admission. Incidentally, all except the latter are routinely collected by the network and will therefore be included in the near future.

Of the 20 criteria required for quality indicators according to QUALIFY [7], the network applies 14 as instructed and 3 in a modified version: Reliability would best be tested using a test-retest or an inter-rater procedure. This is however not possible because of the limited funding available. Instead, we established an algorithm designed to flag data that are selected for a partial test-retest procedure. The other modifications were necessary because of the relatively small collective size in Switzerland where some of the units only have ca. 30 cases per year: The ability for statistical discrimination in QUALIFY requires limits as of which an outcome switches from good to poor quality in order to calculate the minimal amount of patients required by a participating unit to guarantee a secure statistical statement. Such limits are not yet available in neonatology. Since the low collective size in Switzerland cannot be modified and the network does not have the intention to define good or bad quality, but rather to identify possible areas of improvement, we instead optimize statistical reliability by pooling years and optimize finding relevant results by offering the same data for consecutive pooled years in three different collectives (very preterm, very low birth weight and extremely preterm). This way, large and potentially relevant outcomes are sometimes discussed even if they have not yet reached statistical significance. Finally, risk-adjustment has been simplified to reflect only the units’ individual distribution into gestational age groups. Any additional risk-adjustment would stratify the small collectives into even smaller groups making no more statistical sense.

The network omits 3 of the QUALIFY criteria: Sensitivity and Specificity calculation require the presence of gold-standards which have not yet been established for the variables observed in this study. Comprehensibility and interpretability for patients and the interested public have been omitted as we believe the network’s quality cycle to require too much expertise to be distributed to the general public.

### Serviceability for quality improvement

Ellsbury et al. (2010) [10] maintain that despite the complexity of the NICU environment, significant improvements can be accomplished by use of basic QI methodology. The network can provide several aspects of the required methodology postulated by Ellsbury et al. The tools to identify a clinically important and modifiable outcome, the setting for establishing a goal for improvement and the structure for securing a long-time establishment of the change by continuous data collection and review. Hulscher et al. (2013) particularly point out the requirement of latter as they observed that if teams remained intact and continued to gather data, chances of long-term success were higher [21].

The remaining aspects of the methodology required according to Ellsbury et al. (2010) however need to be provided by the units directly: a team that finds the “vital few” causes for the problem according to the Pareto principle (as opposed to the “trivial many” causes) and that is motivated to implementing the change, preferably a system change as opposed to tinkering [10].

Starting from a different vantage point, Lloyd (2010) describes milestones required for reaching quality improvement [22]. The network observes these milestones: The aim of the network’s quality cycle is clearly specified, it follows a concrete concept, the items and how they are measured are well defined, a well-developed data collection plan exists and the data are analysed both statically and analytically. We however prefer Plsek’s p-charts over the run or Shwehart charts proposed by Lloyd due to the latter’s complexity which makes them difficult to program for them to be produced automatically, and because of the limited population size in some of the participating units which would limit the explanatory power of the run or Shwehart charts. Noteworthy however is that Lloyd’s sequence for improvement parallels our proposed quality cycle (Figure 3) if rotated anti-clockwise by 90 degrees. His “act-plan-do-study” translates into our “guideline (plan)-perform (do)-falsify (study)-reform (act)” which again is listed in Ellsbury et al. (2010) congruently as “plan-do-study-act” and is said to be a simple feedback cycle with a long history of successful use in improvement activities in industry and many other fields [10,11].

### Falsification

Kelle et al. (2010) maintain that constant doubt is a basic tenor in evidence based medicine and conclude that this doubt can be used to detect typical misperceptions and erroneous conclusion [23]. Swiss Level III neonatology units apply evidence based guidelines and in centre or multicentre based studies also develop such guidelines using random controlled trials [24]. Under the premises that neither scientific research nor clinical performance are immune to human error, in particular when working with fully established and proven evidence based guidelines, we propose that a long-term constant monitoring of key clinical measurements will help in the establishment of useful guidelines versus ineffective ones because it allows observing the effect of the guidelines on the everyday clinic from a so far un-established vantage point. In other words, we believe constant doubt is a prerequisite for evidence based medicine and therefore its application should be continuously tested (respectively falsified) in order to secure that the knowledge gained by statistical interpretation of probabilities really is a reflection of the true nature of the problem for which the evidence based solution was found. The network’s tool however cannot be seen as a final answer to this dilemma, merely as a step in the direction of accepting the constant doubt.

This is another reason why we maintain that the network’s goal is not to classify good or bad quality but is instead designed to detect possible errors by performing constant falsification. Obviously this is open for improvement by augmenting the range of observed measurements and further refining its methodology.

### Limitations

Statistical discrimination requires large numbers or large differences. Swiss neonatology units offer neither: The units have approximately 30 to 160 cases per year and comparable quality standards. That is why we need to pool years and deviate somewhat from the recommendations made by QUALIFY.

The choice of items measured by the network is so far dependent on routinely collected variables for research. New items can be added but the network has limited itself to performing changes in the data collection only every five years in order not to risk the quality of the data collection of the existing items. Data pooling and the necessity to gather twice the amount of data to perform our reliability exam, result in a productive routine integration of a new item only after 4 years. The waiting however can be shortened, if need be, by replacing the reliability test through a test-retest method. Also, preliminary data can be observed on a unit’s level from the beginning of data collection with limited explanatory power. Nevertheless, due to its complexity, the network’s tool is not very flexible.

As the risk-adjustment for each unit is different, the units’ values cannot be directly compared to each other in the QI chart. We however deem this as irrelevant, as we are interested in each unit’s performance vs. the collective and not in the direct competition between units.

The Swiss Neonatal Quality Cycle is still in its beginning phase. The effects listed at the end of the results section result from preliminary meetings held during the development of the quality cycle. We are currently monitoring the measures undertaken to improve quality in order to be able to concretely report on observable effects over time. But even if we can report on a significant change attributed to the quality cycle, we will not be able to empirically prove that the observed change is in fact caused by the quality cycle, as for instance recommended by Schouten et al. (2008 and 2013) [21,25]. In essence, we can never rule out that other simultaneous changes (such as new medication or evidence based measures) are in fact responsible. The setup does not fulfil the criteria met by controlled trials and has no intention to do so.

## Conclusions

The Quality Cycle of the Swiss Neonatal Network is a helpful instrument to monitor and gradually help improve the quality of care in a region with high quality standards and low statistical discrimination capacity.

## Abbreviations

CPAP: Continuous positive airway pressure; NICUs: Neonatal intensive care units; SMR: Standardized mortality ratio; QI: Quality indicator; CIRS: Critical incident reporting system; PDA: Patent ductus arteriosus; NEC: Necrotizing enterocolitis; ROP: Retinopathy of prematurity; UapH: Umbilical artery pH; PIH: Periventricular-intraventricular haemorrhage; cPVL: Cystic periventricular leucomalacia; PPH: Positive pulmonary hypertension; SNN: Swiss neonatal network; GA: Gestational age; VON: Vermont Oxford network.

## Competing interests

The authors declare that they have no competing interests.

## Authors’ contributions

MA and HUB had primary responsibility for the study design, data acquisition, data analysis and writing the manuscript. TH was involved in research, data interpretation and writing of the manuscript. All authors read and approved the final version of this manuscript.

## Pre-publication history

The pre-publication history for this paper can be accessed here:

http://www.biomedcentral.com/1471-2431/13/152/prepub
